# Moral orientations in psychology: contrasting theoretical perspectives

**DOI:** 10.1186/s40359-019-0287-y

**Published:** 2019-02-22

**Authors:** James C. Wiley

**Affiliations:** Department of Psychology, Carleton University, 1125 Colonel By Drive, Ottawa, Ontario K1S 5B6 Canada

**Keywords:** Relational developmental systems, Trade-off, Life history theory, Morality, Ethics

## Abstract

**Background:**

The relational development systems (RDS) metamodel embodies a newly recognized scientific paradigm that stands in contrast to the nature-nurture split. It suggests that the bidirectional relationship between an organism and its environment must be the central focus of scientific inquiry.

**Main body:**

RDS theorists suggest scientists have a moral obligation to benefit human kind. However, the potential for interventions that appear efficacious to simultaneously instigate an undesirable outcome suggests that moral clarity might not always exist in scientific practice. Contrasting RDS perspectives with life history theory highlights a pertaining disparity in approaches.

**Conclusion:**

While the RDS metamodel posits many premises necessary to contemporary research, it may not yet be pragmatic to impose moral obligation on the sciences.

## Background

The field of psychology is distinguishably compartmentalized, with differing areas of scientific inquiry acting as their own segregated disciplines (e.g., cognitive psychology, health psychology) [1, 2]. However, those studying life-span development have drawn from many areas of scientific inquiry in an attempt to create a globalized understanding of the human experience [3, 4]. This has facilitated the creation of the relational developmental systems (RDS) metamodel [4–7], which constitutes essential criteria for lower-level research models that seek to explain human developmental patterns. While many aspects of the RDS metamodel are of scientific value, some of its propositions lack pragmatism. Specifically, RDS theorists suggest that the sciences have an explicit obligation to benefit the world, which may undermine the potential for positively oriented interventions to facilitate undesirable outcomes. In contrast, life history theory [8] illustrates how an intervention that appears morally righteous can have severe negative consequence for its target population. A better understanding of how positive and negative outcomes relate to each other can create a moral grey area when appraising intervention efficacy. While RDS thought is of great value to researchers, a purely moralistic approach in the sciences does not yet appear pragmatic.

The RDS metamodel attempts to explicitly define the paradigm through which contemporary psychological research is conducted [4]. Thus, it is generally representative of perspectives in psychology and bears similarities with many pertaining theories. RDS proponents seek to outline the boundaries within which contemporary psychological science typically operates [4, 6]. This distinguishes the RDS metamodel from lower level models, in that it aims to make explicit the often implicit assumptions made in psychological research. By pinpointing assumptions that are commonly made across segregated psychological disciplines, RDS theorists hope to promote a more unified understanding of the field as a whole.

In representing contemporary perspectives in psychology, the RDS metamodel stands in contrast to the Cartesian split through which many sciences have previously operated [4, 6]. This former scientific paradigm viewed the variables of nature (i.e., genetic makeup) and nurture (i.e., environment/ecology) as being independent of each other [4, 9]. Hypotheses developed from this perspective assumed that genetics and the environment produced separate effects on the individual. Under this assumption, the individual, their genetics, and their ecology maintained segregated identities [9]. In contrast, contemporary social science perspectives suggest that nature and nurture cannot be understood as separate from one another. In accordance, the RDS metamodel stresses that the inherent qualities of an organism and its environment exert varying influences on each other over time, causing either one to change and cyclically impact the other [4, 6]. Thus, the individual and their context share a bidirectional relationship, which is often represented in the literature using a bidirectional arrow sign (↔). Due to the potential for individual ↔ context relations to vary in manifestation over the passage of time, the opportunity for change across the lifespan is omnipresent [4, 10]. This opportunity for change is termed *plasticity*, and was conceptually developed by scholars who sought to reject perspectives based on genetic determinism: the idea that development is preconceived by an organism’s genetic makeup [2, 4, 8, 11–13]. A large body of research from varying disciplines has shown support for the RDS metamodel and the existence of plasticity across the lifespan [13–16]. Without exemption, the biological sciences suggest that an individual’s environment can regulate their genetic expression [4, 9, 17].

The RDS metamodel is applicable across many disciplines and guides the approaches scientists take when explaining phenomena [4]. While contemporary theories operating within the RDS metamodel might vary in assertions, precision, and subject, they remain united by its overarching premises. For example, Bronfenbrenner’s [18] theoretical discussion of human ecological systems examines the individual’s mutual relationship with their entire ecology, ranging from their school, place of work, family members, and friends (i.e., microsystem and mesosystem), to the transcendent societal influences of the media, the law, and the political climate (i.e., exosystem). The way in which all these factors cyclically influence each other is synthesized by a consideration of the entire culture that the individual is situated in (i.e., the macrosystem), as well as the idiosyncrasies of the timing of their experiences and livelihood (i.e., chronosystem). Alternatively, a more concrete application of the RDS metamodel is presented by Lynch et al. [19], who examined the reciprocal relationship between individual youths and a community oriented program to which they belonged (i.e., the Boy Scouts of America). Examining Boy Scout groups and their members, measurements of individuals’ involvement with their particular group were taken, and from these an overall score of involvement was conceived for that group. This method allowed for individual ↔ group relationships to be gleaned. In particular, Lynch et al. [19] focused on how a particular psychological construct of interest (i.e., character development) and its pertaining facets varied as functions of individual ↔ group involvement. Overall, high group engagement was found to amplify the positive impact of high individual engagement on character development. By considering the intertwined nature of the individual and the group, Lynch et al. [19] provide a wholesome depiction of the relationships between variables of interest.

The above premises appear well accepted across the human sciences [4, 11, 14]. However, the RDS metamodel also adheres to an ethical obligation to facilitate positive human development [4, 20]. This perspective bears a degree of controversy when contrasted with viewpoints in medicine and public health [8]. Lerner et al. [4] state that RDS based research is capable of conjunctly explaining and enhancing developmental outcomes. Accompanying the paradigm shift away from the Cartesian Split has come a notion that scientific inquiry will no longer stand segregated from scientific application [4, 12]. In other words, scholars will cease to invest in the divide between purely inquisitive research and applied research. This idea has been further stressed by other developmental scholars, who suggest that a scientific field capable of improving “the course of human development is ethically obligated to do so” (Fisher, Busch-Rossnagel, Brown, & Jopp [21]; Lerner & Overton [20] as cited by Lerner et al. [4] p. 96). Coinciding with this, psychological science in general totes a high degree of supposedly inherent moral validity, with researchers consistently describing their work as having some benefit to the world at large. Positive or desirable variables might include the acquisition of happiness [22, 23], high academic achievement [24], a desirable occupation [25, 26], or recovery from mental illness [27–29]. While moral trajectories are covertly implied in most psychological research, RDS theorists overtly push for them to be treated as inseparable from science as a whole.

## Main text

### Coinciding benefits and detriments

The historical context that contemporary psychological science has evolved from distinctly illustrates the importance of ethical consideration and conduct (e.g., Baumrind [30], Zimbardo [31]). While the entirety of this history has played a part in shaping modern scientific practice, RDS theorists take particular distaste with the idea of genetic determinism [4, 13, 14]. This contention is well placed, considering deterministic principles have been used nefariously as a pseudoscientific justification for discriminatory beliefs [13, 32]. For instance, the false claim that a particular gene codes for intelligence has been iterated with the intent of pinpointing racial demographics that are prone to stupidity [32, 33]. While not its sole purpose, the RDS metamodel does well to illustrate the vast potential that all organisms have for achieving varying outcomes across the life-course. Being a metatheory that is broadly applicable to all human sciences, it does not permit the existence of genetically deterministic principles. Stemming from this, Lerner et al. [4] provide a core justification for the inseparability of morality and science: that the vast interconnection between all components of person ↔ context relationships inevitably means some part can be manipulated to facilitate a benefit. Organisms are not fixed to a particular life trajectory and can be changed for the better no matter how impossible their situation may appear.

Highlighting the vast potential for science to facilitate positive human development has distinct merit. However, RDS theorists may underestimate the potential for beneficial interventions to simultaneously instigate undesired consequences, adding a degree of moral ambiguity to their administration. While it is possible that some obscure intervention exists that can benefit even those in absolutely dire conditions, the opposite has also been observed, where an overtly beneficial intervention also facilitates harm [8]. In their paper discussing evolutionary approaches to the study of public health, Wells et al. [8] highlight research from Gibson and Mace [34] examining the impact that the installation of water taps had on a community situated in Arsi, Southern Ethiopia. Preceding installation, women were often responsible for transporting water back to their community in clay pots. Water tap installations would eliminate this long and cumbersome task from their daily activities. While one might assume that this highly desirable change in lifestyle would permit an increased investment in childcare, resulting in better quality of life for children, Gibson and Mace [34] found something quite different. Water tap installation was related to malnutrition in children, while also predicting improved fertility in women rather than improved health. Interpretations suggest that the already scarce resources available to households were being stretched between larger numbers of offspring, facilitating this decline in children’s nutrition.

While ethically it is desirable to reduce the manual labour required for families to obtain necessary resources, this was only realized with worsened health conditions for a portion of the target population. Lerner et al. [4] explicitly highlight the vast potential that interventions have for promoting positive outcomes. In contrast, Wells et al. [8] suggest that interventions facilitating benefits can also incur some obscure negative trade-off. This suggests that moral grey areas may arise when scientific advancement is used towards positive ends. A more complete understanding of intervention efficacy may bring similar moral values into conflict. Scientists may be forced to choose one result over another, despite both being desirable for target populations.

### Life history theory

These realizations are situated within life history theory, which posits that humans have evolved to maximise reproductive success rather than physical health [8]. By observing how energy provided via intervention is invested into either health or fertility, refinements can be made that better achieve desired outcomes. Life history theory distinguishes two crucial concepts: reaction norms and trade-offs. Reaction norms bear a degree of synonymy with plasticity: they compose the spectrum of possible traits and behaviours arising from a singular genetic composition. Reaction norms represent how differing contexts permit variation in outcomes for genetically similar organisms. As discussed above, trade-offs occur when a benefit to an organism is accompanied by a detriment. For instance, health is often in competition with fertility over resources available to the individual. Administering a benefit to a population could facilitate reassignment of already available resources to reproduction rather than simply increasing the resources invested into maintaining health. Life history theory warns that interventions must first consider the resources available to a target population before providing new assets. A provided benefit is unlikely to have an independent impact, and may also facilitate harm. While it is crucial that researchers understand these trade-offs, they may not always permit moral clarity in practice.

Many of these realizations are not new to RDS thought [4]. However, life history theory does distinguish a component unmentioned by RDS theorists: that the potential for positive ↔ negative trade-offs may be accompanied by moral ambiguity [8]. While the vast complexity of person ↔ context relations suggests there is always some obtainable positive outcome [4], by the same logic there is always potential for some unobserved harm. Considering the aggressive orientation that psychologists take in promoting their work as being beneficial to the world, there may be a failure to recognize negative trade-offs accompanying their provided benefits [35]. Nesse [35] illustrates this issue well in his discussion of anti-depressants and their over-administration. While perhaps successful in treating depression, depression itself is an evolutionary reaction generated within the individual ↔ environment relationship, and may serve the individual some benefit. Nesse [35] stresses that the treatment of depression should not be considered in binary fashion, where anybody with clinical level symptomology is prescribed a medication regimen. The occurrence of depression is complex and can be treated in many ways, whether pharmacological or psychological. Nesse [35] suggests that treatments should aim to improve individuals’ long-term wellbeing rather than to evict immediate issues. If a patient has a condition where they require pharmacological intervention to alleviate depressive symptoms, then anti-depressants are optimal for achieving this goal. However, if an individual with a relatively normal psychological pathology experiences a depressing life event, their expression of depressive symptoms is not immediately cause for concern. They are in fact exhibiting a healthy evolutionary prognosis. Experiencing these intense negative feelings in their immediate situation could help them avoid future contexts that will elicit the same problems. If intervention administrators consider it their responsibility to provide a net benefit to their subjects, it is essential that they consider more than just the target variable when appraising intervention efficacy [8]. While alleviating immediate suffering has a moral appeal, it is debatable as to whether it is the best course of action in all situations [35]. It should be considered that the goal of reducing depressive symptoms is not really an optimal objective that considers all the idiosyncrasies of the condition. Equally sub-par is the goal of improving only the availability of water in a community deprived of a multitude of resources [34].

### Observable trade-offs

Life history theory mandates researchers to integrate evolutionary principles into their practice [8]. However, positive ↔ negative trade-offs are already an accepted part of medical and physical health sciences. For example, treatments aimed at preventing breast cancer reoccurrence are often accompanied by a plethora of harsh side-effects [36]. Pertaining toxicities include osteoporosis, hot flashes, sexual dysfunction, infertility, and depression. Additionally, these treatments must be endured for 5 to 10 years and do not guarantee prevention. Cancer interventions are akin to gambling strategies: even if possessing an effective method one will still lose on occasion. Unfortunately, oncologists balancing the probability of death with their patients’ suffering is a rather dark gamble. Breast cancer researchers have at their disposal the tools necessary to optimise human suffering and death by tuning treatment administration. All that remains is to decide on the perfect suffering-to-death ratio. While it is easy to rule out the extremities of the ratio (i.e., minimal human suffering and maximised death, maximized human suffering and minimal death), one cannot morally justify a distinct optimization. At face value there is no real grey area when it comes to treating breast cancer, but practitioners still face moral dilemmas that have no obvious solution.

While less morally ambiguous due to the known efficacy of treatment, insulin regimens for diabetes are similarly accompanied by harsh side-effects [37]. For instance, insulin lowers patients’ blood sugar levels, potentially leading to dysphoria, loss of consciousness, lowered cognitive and motor function, or death [38]. While improving a patient’s understanding of their illness can improve treatment adherence, it remains difficult to balance immediate suffering due to side-effects with future health and prolonged life [37, 39]. Diabetic patients can suffer a variety of health complications for failing to control their blood sugar levels, such as retinal damage, heart failure, or kidney failure [38]. However, higher blood sugar will facilitate elevated mood. When contrasted with the dysphoria accompanying insulin injections, it can be difficult for patients to understand and for doctors to justify the benefits of treatment adherence. Although the consequences for failing to treat diabetes are debatably severer than the side-effects of treatment, justifying this trade-off to patients remains a challenge for practitioners [37].

In medicine it is commonly assumed that all interventions balance negative side-effects with efficacious treatment [40]. No intervention is perfect, and inevitably causes harm in some regard. Medical practitioners are required to help their patients manage these trade-offs and judge if the harm incurred is greater than the benefit received. While psychological interventions influence recipients more abstractly, it seems inevitable that they also facilitate positive ↔ negative trade-offs [35]. Additionally, physical health consequences can be measured in concrete fashion and are more limited in scope, whereas the number of psychological factors and their varying facets cannot be so easily observed and summarized. While less integral to the discipline, trade-offs are similarly recognizable in psychology. For instance, individuals possessing psychopathic characteristics rate higher in desirable business skills [41]. Those who consistently project their true personalities have been found to be less successful in their occupations than those who meld their personalities to best suit immediate social settings (i.e., high self-monitors; [42]). While conformity yields a variety of social benefits [43], related detriments are famous in experimental psychology (e.g., Milgram [44], Zimbardo [31]). Contemporary observations continue to yield interesting findings on this front. For instance, children’s moral judgment can be negatively influenced by peers’ opinions and behaviour [45]. In adolescence the presence of peers amplifies the likelihood of risk-taking behaviour [46]. Still other areas of psychology reflect trade-offs. Facilitating academic success in youth is considered highly desirable [47]. However, studies on education related anxiety suggest that high achieving students are often substantially more anxious than their peers [48, 49]. Relationship commitment appears crucial to couples’ relationship quality, which a variety of programs seek to promote [50]. However, the former variable also positively relates to a victim’s decision to stay with their abusive spouse [51, 52]. Contradicting moral orientations within these bodies of literature have facilitated a more complete understanding of their subjects. While some have higher degrees of moral ambiguity than others, each one illustrates how interventions tweaking one component of the person ↔ context relationship for the better could be accompanied by a negative outcome.

The prevalence and importance of positive ↔ negative trade-offs in contemporary human sciences brings into question the pragmatism of the claim that purely positive and morally righteous interventions can be sought after. While one can certainly be optimistic that some beneficial approach exists for every situation [4], one can be equally pessimistic that such an approach may facilitate an unknown harm [8, 35]. From an abstract orientation there is equal potential for benefit and harm when tweaking components of the person ↔ context integrated system. However, empirically it has been accepted that positive ↔ negative trade-offs are unavoidable in some practices [8, 40]. Perhaps this idea warrants further attention in psychological research, where it may be easy to overlook potential detriments [35]. As researchers consider these problems more thoroughly their ability to arrive at moral conclusions about them may advance. However, as of current it is difficult to see pragmatic value in the claim that purely positive results are always obtainable in all situations.

## Conclusions

The RDS metamodel represents significant advancements for psychological research as a whole [4]. It has brought together many of the compartmentalized sub-disciplines of the field and embodies a new understanding of the nature-nurture relationship. Despite these benefits, RDS theorists adhere to an ethical obligation to better the world, which may not be a pragmatic scientific orientation. Wells et al. [8] suggest that a complete understanding of an issue could highlight negative consequences stemming from morally justified interventions. Such trade-offs are commonly accepted in medical and physical health disciplines [8, 40] and may warrant further attention in psychological science. While ethical conduct is an essential part of contemporary psychology, this does not eliminate the natural prevalence of morally ambiguous situations arising in research and practice.

## Abbreviation

RDS: Relational development systems
